# Serum Metabolomic Profiles in Critically Ill Patients with Shock on Admission to the Intensive Care Unit

**DOI:** 10.3390/metabo13040523

**Published:** 2023-04-05

**Authors:** Aurélie Thooft, Raphaël Conotte, Jean-Marie Colet, Karim Zouaoui Boudjeltia, Patrick Biston, Michaël Piagnerelli

**Affiliations:** 1 Intensive Care, CHU-Charleroi, Université Libre de Bruxelles, 140, Chaussée de Bruxelles, 6042 Charleroi, Belgium; patrick.biston@chu-charleroi.be (P.B.); michael.piagnerelli@chu-charleroi.be (M.P.); 2 Laboratory of Human Biology and Toxicology, Research Institute for Health Sciences and Technology, University of Mons, 7000 Mons, Belgium; raphael.conotte@umons.ac.be (R.C.); jean-marie.colet@umons.ac.be (J.-M.C.); 3 Laboratory of Experimental Medicine, ULB 222 Unit, Université Libre de Bruxelles, CHU-Charleroi, 6110 Charleroi, Belgium; karim.zouauoi@chu-charleroi.be

**Keywords:** gluconeogenesis, proteolysis, lipolysis, septic shock, prognosis

## Abstract

Inflammatory processes are common in intensive care (ICU) patients and can induce multiple changes in metabolism, leading to increased risks of morbidity and mortality. Metabolomics enables these modifications to be studied and identifies a patient’s metabolic profile. The objective is to precise if the use of metabolomics at ICU admission can help in prognostication. This is a prospective ex-vivo study, realized in a university laboratory and a medico-surgical ICU. Metabolic profiles were analyzed by proton nuclear magnetic resonance. Using multivariable analysis, we compared metabolic profiles of volunteers and ICU patients divided into predefined subgroups: sepsis, septic shock, other shock and ICU controls. We also assessed possible correlations between metabolites and mortality. One hundred and eleven patients were included within 24 h of ICU admission, and 19 healthy volunteers. The ICU mortality rate was 15%. Metabolic profiles were different in ICU patients compared to healthy volunteers (*p* < 0.001). Among the ICU patients, only the subgroup of patients with septic shock had significant differences compared to the ICU control patients in several metabolites: pyruvate, lactate, carnitine, phenylalanine, urea, creatine, creatinine and myo-inositol. However, there was no correlation between these metabolite profiles and mortality. On the first day of ICU admission, we observed changes in some metabolic products in patients with septic shock, suggesting increased anaerobic glycolysis, proteolysis, lipolysis and gluconeogenesis. These changes were not correlated with prognosis.

## 1. Introduction

Critically ill patients admitted to the intensive care unit (ICU) have an altered metabolic response following the development of inflammation, anaerobic metabolism, hormonal variations and profound changes in energy balance, favoring a catabolic state [1]. These complex metabolic modifications include activation of hepatic gluconeogenesis, increased proteolysis with a release of amino acids and increased lipolysis, which evolve quickly over time.

Metabolomics—defined as the quantitative measurement of the dynamic metabolic response [2] to pathophysiological conditions—can provide metabolic profiles by the simultaneous analysis of multiple metabolites to provide a precise picture of (in)active metabolic pathways. Several metabolomic studies in animals and humans identified metabolic profiles that can differentiate subjects with a large range of diseases, including trauma [3], acute respiratory distress syndrome (ARDS) [4] and sepsis [5], from healthy individuals. The major changes as a result of the inflammatory processes associated with all of these pathologies were a switch toward anaerobic metabolism and stimulation of gluconeogenesis.

Interestingly, several human studies have shown an association between the metabolic profile at ICU admission and prognosis [6]. In trauma patients, Cohen et al. [7] showed different lipid profiles on ICU admission in survivors and non-survivors. In patients with acute lung injury (ALI), Stringer et al. [8] reported higher plasma levels of phosphatidylserine and glutathione compared to healthy volunteers, and that was correlated with the Acute Physiology Score (APS). Mickiewicz et al. [9] showed a difference in the metabolic profiles of patients with septic shock compared to non-infected ICU patients. These differences mostly involved profiles implicated in energy metabolism, and certain metabolic differences were correlated with outcomes.

Rapid identification and management of patients are important in optimizing survival in sepsis [10,11]. Identifying disease severity markers within the metabolic profiles of patients with sepsis could help to develop new therapeutic pathways or personalized treatments. The aim of this work was to identify ICU admission metabolic profiles that could serve as prognostic markers in different groups of critically ill patients.

## 2. Materials and Methods

After approval by the local ethics committee (P14/50_03/12), this study was conducted over a one-year period in a medico-surgical 24-bed ICU at CHU-Charleroi, Belgium. Written informed consent was obtained from all subjects or their surrogate decision-maker.

### 2.1. Patients and Healthy Volunteers

During the first 24 h following ICU admission, all adults whose ICU stay was expected to be at least 48 h and who had an arterial catheter in place were considered for inclusion. Patients admitted for post-operative monitoring for ≤24 h, who were pregnant or who received a blood transfusion, were excluded. At inclusion, ICU patients were divided into four groups: sepsis (suspected or proven infection, with general, inflammatory and/or hemodynamic signs of sepsis and signs of organ failure [12]); septic shock (sepsis with hypotension requiring norepinephrine after intravascular filling); another shock (circulatory shock requiring norepinephrine with an origin other than infection); and ICU controls (ICU pathologies not included in the previous groups, e.g., decompensated chronic obstructive pulmonary disease, drug intoxication, trauma, stroke, cerebral trauma, …). Age, sex and co-morbidities (chronic arterial hypertension defined as arterial pressure > 140/90 mmHg, chronic renal failure defined by a glomerular filtration rate < 60 mL/min/1.73 m^2^, diabetes mellitus) were recorded for all patients; body mass index (BMI), sequential organ failure assessment (SOFA) [13] and acute physiology and chronic health evaluation (APACHE) II [14] scores were calculated. The ICU-, 28-day- and 90-day mortality rates were noted.

Nineteen healthy volunteers (6 males and 13 females, aged 37 ± 12) not taking any drugs or medication were also included as control subjects.

### 2.2. Metabolomic Studies

An arterial blood sample of 6 mL was drawn (BD Vacutainer tube for sera) and immediately centrifuged at 1731 rcf for 15 min at 4 °C. The serum was removed and conserved in cryotubes at −80 °C for further metabolomics analysis, performed in the Human Biology and Toxicology Unit of the University of Mons, Belgium.

### 2.3. Proton Nuclear Magnetic Resonance (^1^H-NMR)

Five hundred μL of serum was analyzed before and after filtration to eliminate molecules with a molecular weight superior to 3 kDa; 250 μL of a phosphate buffer solution (0.22 M Na_2_HPO_4_/0.04 M NaH_2_PO_4_, pH7.4) was added. A 550 μL sample of the final solution was removed and transferred to a 5 mm NMR tube containing 50 μL of 3 mmol TSP (acid 3-(trimethylsilyl)propionate-2,2,3,3-d_4_) as an external reference. The final sample was analyzed by *^1^H-NMR* spectroscopy on an Avance 500 Bruker spectrometer (11.8 Tesla corresponding to a 500 MHz proton Lamor frequency). A presaturation pulse sequence was used to minimize the contribution from water proton resonance. For each acquisition, a total of 32 scans were obtained. After Fourier transformation (FT) of the free induction decay (FID) to obtain signal intensities as a function of the resonance frequencies (chemical shifts, δ), and line broadening, the spectra were baseline- and phase-corrected. The δ of the TSP single resonance was arbitrarily placed at 0.00 ppm, which served to calibrate the other peaks. The region between 0.08 and 10.00 ppm was then divided into 248 sub-regions (also called descriptors), each of 0.04 ppm width, for which the integral of the area under the peaks (AUC) was calculated using the MestReNova software (MestreLab Research, Santiago de Compostela, Spain). The spectral sub-region between 4.5 and 5 ppm was excluded prior to analysis to remove any possible residual water peak.

To exclude any potential bias in the metabolic profiles of ICU patients from drug-related compounds, we also established the NMR profiles of medications commonly used in the ICU (catecholamines, sedatives, analgesics, antipyretic drugs, antibiotics).

### 2.4. Biological Data and Treatments

At the same time as the sample for the metabolic profiling, the following parameters were also recorded: biological by another sample (leukocyte, platelet and hemoglobin counts, blood glucose and lactate levels, urea, creatinine, bilirubin, albumin and C-reactive protein concentrations) and hemodynamic (heart rate, mean arterial pressure) parameters, temperature, and arterial blood gases to determine PaO_2_/FiO_2_. We also recorded doses of vasopressor and sedative agents.

## 3. Statistics

Data were analyzed using SIMCA-P+ version 12.0 (Umetrics, Umeå, Sweden) and SigmaStat version 12.0 (Systat Software Inc., San Jose, CA, USA). Continuous variables are presented as mean ± standard deviation if normally distributed and as median and interquartile range if not normally distributed. Categorical variables are presented as numbers and percentages. A multivariable analysis was performed using two methods [15]: first, an unsupervised method (principal component analysis [PCA]) was applied to the binned data set to identify any possible outliers, and then a supervised method (discriminant analysis [DA] of [orthogonal] partial least squares ([O]PLS)) was used to identify potential clustering in the dataset. The models obtained from the supervised method were characterized by cross-validation (CV). We obtained R^2^ and Q^2^, indicators of the power and the quality of the predictive model, respectively. We applied a variance analysis using ANOVA. The VIP method (variable importance in projection) was also used to identify variables that contributed to the model (only variables with a VIP > one were considered significant). The first component (PC1) was used to select the VIPs. A Kruskal–Wallis test followed by a Dunn correction was used to analyze differences in metabolites between sub-groups. The correlation was analyzed using Spearman’s test. A *p*-value less than 0.05 was considered significant.

## 4. Results

### 4.1. Biological Data and Treatments

During the study period, 111 consecutive patients were included: 37 with sepsis, 39 with septic shock, 6 with other shock and 29 ICU control patients. A first PCA identified an outlying patient with a high ethanol peak that was confirmed in the blood sample. This patient was therefore excluded from further analysis. The clinical data of the remaining 110 patients are shown in Table 1. ICU-, 28- and 90-day mortality rates were 15, 25 and 29%, respectively. As expected, ICU severity scores were higher in patients with shock than in other patient groups (Table 2); these patients also had higher blood lactate concentrations (Table 3). C-reactive protein concentrations were higher in patients with sepsis and septic shock than in other patients (Table 3). Hemodynamic variables and treatment are presented in Table 4.

### 4.2. Metabolomic Studies

**Figure 1:**
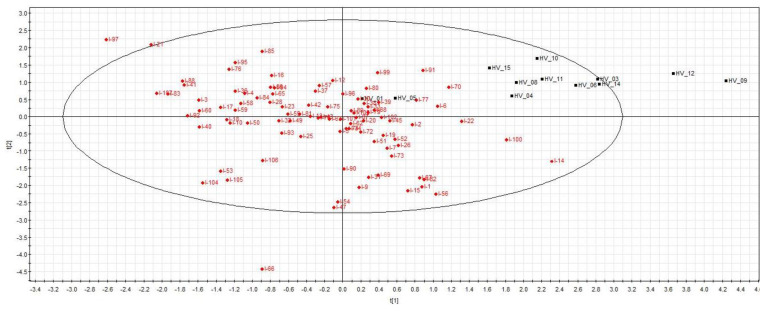
Scores plot (healthy volunteers versus ICU patients). The abbreviations HV represent healthy volunteers; the abbreviation I represent ICU patients. PLS-DA shows a separation between healthy volunteers and ICU patients (R^2^X = 0.27; R^2^Y = 0.62; Q^2^ = 0.44).

**Figure 2:**
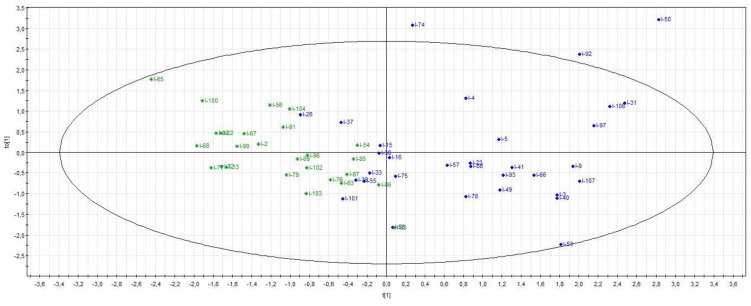
Scores plot (septic shock versus ICU control group). Septic shock patients (in blue) versus ICU control patients (in green). The supervised OPLS-DA method shows a significant difference between septic shock patients and ICU control patients (CV-ANOVA, *p* = 0.006).

**Figure 3:**
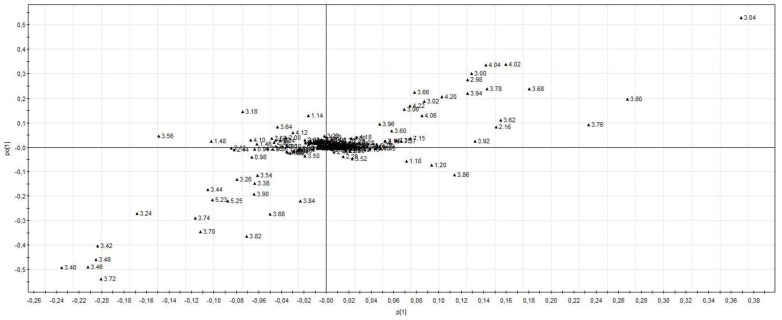
Loadings plot (septic shock versus ICU control group). Different expressions of metabolites between septic shock and ICU control group (R^2^X = 0.53; R^2^Y = 0.58; Q^2^ = 0.29).

**Figure 4:**
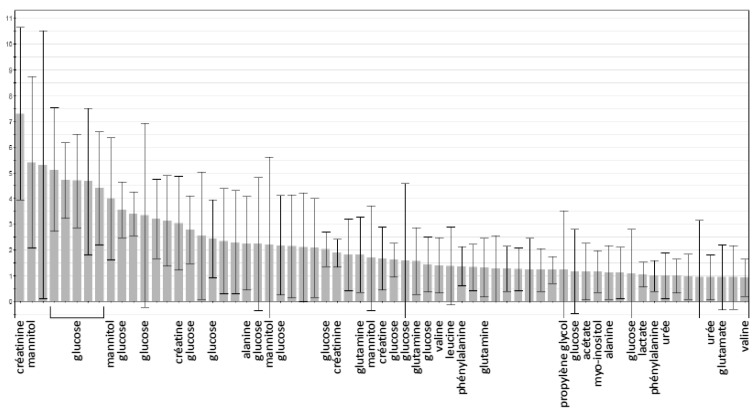
VIP plot (septic shock vs. ICU control group). Identified metabolites considered as relevant when VIP > 1: creatine, creatinine, urea, myo-inositol, phenylalanine, mannitol, alanine, valine, glutamine, glutamate, leucine.

The supervised PLS-DA analysis (R^2^X = 0.27; R^2^Y = 0.62; Q^2^ = 0.44) showed significant separation between healthy volunteers and all ICU patients (CV-ANOVA, *p* < 0.001) (Figure 1). Among the ICU sub-groups, PCA showed a trend in the separation of patients with and without septic shock, which was not confirmed with the supervised analysis. There was also a separation of septic shock and ICU control patients, which was significantly different from the supervised OPLS-DA method (R^2^X = 0.53; R^2^Y = 0.58; Q^2^ = 0.29) (CV-ANOVA, *p* = 0.006) (Figure 2). From the corresponding loadings plot, we identified different expressions of metabolites between these two groups (Figure 3; Supplementary Materials Figures S1 and S2). Using the VIP method, we identified the relevant factors and observed higher levels of creatine, creatinine, urea, myo-inositol, phenylalanine, 3-hydroxybutyrate and mannitol in the septic shock compared to the ICU control group and lower levels of alanine, valine, glutamine, glutamate and leucine (Figure 4).

The quantitative AUC analysis, normalized to the reference resonance, showed significant differences between the sub-groups for several metabolites (Table 5). There were no correlations between these metabolites and length of ICU or hospital stay or mortality at day 28 or 90 (Table 6).

In the multivariable analysis in the supervised model, there were no differences between ICU survivors and non-survivors.

Of the drugs used in the ICU, one peak in the region of 1.40–1.44, corresponding to the piperacillin-tazobactam spectrum, was relevant in the analysis and was significantly higher in patients with septic shock than in ICU control or other shock patients.

## 5. Discussion

Our study confirms the existence of different metabolic profiles in healthy volunteers and ICU patients and in patients with septic shock compared to other ICU patients. However, these differences were not correlated with outcomes.

The patients with septic shock had the greatest changes in energy metabolism. We observed a significant increase in lactate and pyruvate, reflecting anaerobic glycolysis leading to reduced ATP synthesis [16]. However, increased lactate concentrations may be beneficial as a source for hepatic gluconeogenesis through an accelerated Cori cycle [17]. Proteolysis was also increased in patients with septic shock, as shown on the VIP plot, with a change in levels of several amino acids and high urea levels, suggesting increased amino acid turnover. This increased proteolysis has already been described in the early stage of infection [6,9,18].

Phenylalanine, which was significantly higher in patients with sepsis and septic shock than in ICU control patients, can form fumarate after oxidation, which supplies the Krebs cycle. Su et al. also reported the same results in severe sepsis compared to sepsis, suggesting that phenylalanine is also a marker of disease severity [19]. The higher urea reflects a high level of amino acid degradation, releasing ammonium and carbonated molecules by the liver, which can be either converted to glucose or enter the Krebs cycle. We also observed an increase in creatine—and its degradation product, creatinine—which is a main source of ATP at the muscular level, as already reported in animal models of sepsis [20] and in humans [9]. Indeed, we observed some similarities between the NMR-based metabolic profiles and the normal ranges for blood values. The significant changes observed for lactate, urea and creatinine were all confirmed by conventional serum analysis.

We expected lipolysis to be activated during sepsis in relation to adrenergic stimulation of lipoprotein lipase as described by Kopterides et al. [21], releasing glycerol (a substrate for gluconeogenesis during a period of starvation) and free fatty acids (FFA) (energetic sources and precursors of ketogenesis). However, there were no significant differences in glycerol or ketones bodies (acetone, acetate and 3 beta-hydroxybutyrate) across the groups in our study (Table 5). There are several possible explanations for this finding: first, we filtered the samples to eliminate large molecules that could disrupt the spectrum, and it is possible that some of the lipids were removed during this phase and, therefore, not analyzed. Second, during the early phase of inflammation, as in our study, the contribution of lipids to energetic spending is small, becoming larger over time [1]. Nevertheless, we observed that carnitine (the co-factor associated with acyl-CoA for transport of long-chain carbon FFA across the external mitochondrial membrane) was significantly increased in septic shock patients, but acetylcarnitine (an acylcarnitine corresponding to an inter-membrane form of FFA) was not. This observation may suggest a blockage of the beta-oxidation activity in these patients in the early stage of infection. These results are in contradiction with those of Mickiewicz et al. [22] and Chung et al. [23], who reported a significant increase in acetylcarnitine, suggesting enhanced conversion of increased free carnitine and acetyl CoA concentrations into acetylcarnitine via carnitine acetyltransferase [24]. Changes in metabolic profiles over time may explain these contrasting results.

As has already been observed in several studies [7,8], we also found significantly higher levels of myo-inositol in patients with septic shock. This compound has an important role in intracellular messaging in several tissues, including endothelial cells, adipocytes and the neurological system. Sotoda et al. [25] showed a decrease in myo-inositol incorporation in aortic endothelial cells in rats after lipopolysaccharide (LPS) injection. This decrease may contribute to hypercontractility of the smooth muscle fibers and contribute to the hypotension observed during shock. Recently, Sjöberg et al. [26] proposed myo-inositol as a predictive marker of prognosis after subarachnoid hemorrhage, and Stringer et al. [8] observed a significant correlation between myo-inositol and APS in patients with sepsis-induced ALI, suggesting that this metabolite could be considered as a marker of tissue damage.

In our study, there was a significantly higher concentration of mannitol in the patients with septic shock than in other patient groups. To our knowledge, mannitol is not produced by human metabolism. Nevertheless, this finding has also been reported in other studies. Seymour et al. [27] reported a greater concentration of mannitol in non-survivors of pneumonia than in survivors and, in a rodent model, D’Alessandro et al. [28] reported higher mannitol levels in animals with hemorrhagic shock than in controls. They suggested that this increase was probably due to translocation from the intestinal microbiota. Ethanol is another possible marker of the microbiota, but there were no significant differences in ethanol levels across our study groups. Ethanol was visualized in the spectrum of septic shock patients in the study by Mickiewicz et al. [9] and was significantly higher in non-survivors than in survivors in the study by Garcia-Simon et al. [29].

We identified an unknown metabolite in the region of 1.40–1.44 ppm, which was significantly higher in the septic shock compared to the ICU control patients. Interestingly, this peak was also visible in other studies: in urine samples of patients with sepsis and septic shock in the study by Garcia-Simon et al. [29] and in patients with septic shock in the study by Mickiewicz et al. [9]. After analyzing several drugs commonly used in ICU, this peak was found to match the spectrum of piperacillin-tazobactam. We tested several commonly used drugs but did not identify other corresponding peaks in our patients. This process is difficult because drugs are metabolized in vivo, and some unidentified peaks may be from metabolites of medications and not from the drugs per se.

By contrast with our results, some other studies have found a correlation between some metabolites and patient prognosis and were able to construct mortality models using metabolomic profiling. In two studies, Mickiewicz et al. [9,30] demonstrated a good separation between survivors and non-survivors in their model. This predictive model was established in selected patients of similar ages and sex and not using a correlation test as in the present study, which may explain the different results. Moreover, some metabolites were not identified, and correlations were therefore not tested. In trauma, Mao et al. [31] discriminated patients with organ failure from those with non-complicated trauma by metabolomic profiling. Nevertheless, the differences were only based on a prediction model. Although they had a more homogeneous population than we did, Seymour et al. [27] were unable to construct a prognostic model from metabolic profiles in patients with community-acquired pneumonia.

Our study has several limitations. First, our population is heterogeneous in relation to the site of infection but also to the evolution of the pathology. Although all patients were included during the first 24 h of their admission, the hemodynamic parameters show that our population was already resuscitated and stable. Moreover, patients arrive at the hospital and at the ICU at different moments after the onset of their pathology. Analysis of the time course of the metabolic response could be interesting in this respect. Second, we analyzed serum. A majority of the published studies have been performed on urinary samples, and comparisons are, therefore, difficult. We chose to perform the study on blood rather than urine because patients in shock may be anuric or on dialysis, making urine collection impossible. Future studies should analyze and compare both blood and urine [32,33]. Moreover, Stringer et al. [34] showed significant differences in metabolic profiles from serum and total blood of healthy volunteers. One of the reasons for this observation may be the delay before centrifugation and red blood cell hemolysis, releasing glycolysis metabolites. Nevertheless, the differences in the study by Stringer et al. [34] were only significant when the samples were processed beyond 180 min, and we centrifuged our blood samples after a maximum of 30 min. Third, we studied metabolomics by NMR spectroscopy and not by mass spectrometry. The advantages of NMR are the rapidity of the technique and the fact that the sample can be used several times. Nevertheless, mass spectrometry is more sensitive. Using both techniques together would give a complete cartography of the metabolism [35]. Fourth, because of the small number of patients included in each group and in heterogeneous clinical situations (ICU controls or different types of shocks) and because it does not allow us to perform statistical analyses such as logistic regression or multivariate analyses, this study is exploratory and does not allow us to draw definitive conclusions. Other studies, including a larger number of patients, are necessary. Fifth, we mainly identified metabolites that we could measure using classical technics. Several peaks remain unidentified, and our metabolomic databases need to be expanded.

## 6. Conclusions

There are considerable alterations in metabolism in septic shock patients at ICU admission. These changes include an increase in anaerobic glycolysis, proteolysis, lipolysis and neo-glucogenesis from lactate, phenylalanine and glycerol. Nevertheless, these differences are not correlated with patient prognosis. Critically ill patients are a very heterogeneous patient population. The contribution of metabolomics could allow the development of new biomarkers correlated to the severity of the disease or organ dysfunction and evolving with time. This would allow personalizing treatments according to the patient’s sub-phenotype.

## Figures and Tables

**Table 1:** Clinical data for all patients (n = 110).

Parameters	Value
Age (years)	67 ± 14
Male/Female	58/52
BMI	27.4 ± 7.3
SOFA Score	6 ± 3
APACHE II Score	20 ± 9
Groups	
Sepsis, n (%)	37 (34)
Septic shock, n (%)	39 (35)
Cardiogenic or obstructive shock, n (%)	6 (5)
ICU controls, n (%)	28 (26)
Comorbidities	
IDDM, n (%)	14 (12)
NIDDM, n (%)	19 (17)
Chronic arterial hypertension ^a^, n (%)	62 (56)
Chronic renal failure ^b^, n (%)	23 (21)
Length of ICU stay (days)	8 ± 6
ICU mortality, n (%)	16 (15)
28-day mortality, n (%)	28 (25)
90-day mortality, n (%)	32 (29)

All data are given as mean ± standard deviation. BMI: Body mass index; SOFA: Sequential Organ Failure Assessment; APACHE II: Acute Physiology and Chronic Health Evaluation; IDDM: insulin-dependent diabetes mellitus; NIDDM: non-insulin-dependent diabetes mellitus. ^a^ Chronic arterial hypertension: blood pressure >140/90 mmHg (European Society of Cardiology); ^b^ Chronic renal failure: glomerular filtration rate < 60 mL/min/1.73 m^2^ (MDRD).

**Table 2:** Clinical data for each patient group (ICU control group, sepsis, septic shock, other shock).

	ICU Control Group (n = 28)	Sepsis (n = 37)	Septic Shock (n = 39)	Other Shock (n = 6)	*p* Value
Sex (F:M)	14:14	10:27	20:19	1:5	0.07
Age	60 [48–74]	65 [57–75]	69 [62–80]	78 [53–88]	0.13
BMI	27.5 [25–30.8]	27.9 [22.4–33.0]	26.4 [21.8–31.1]	24.4 [22.9–32.7]	0.95
ICU days	6 [4–7) ]€	6 [4–8] €	9 [6–12]	3 [3–7] €	0.005
SOFA	4 [2–7] €	3 [3–5] €	9 [7–10]	7 [5–8]	<0.001
APACHE II	17 [14–22]	16 [11–21 ]% €	22 [7–30]	26 [23–30]	<0.001
Arterial hypertension, n	17	24	19	2	0.32
CRF, n	4	9	8	2	0.67
IDDM, n	4	6	3	1	0.7
NIDDM, n	3	11	5	0	0.09
Oral nutrition, n	5 $	17 €	6	1	0.012
Enteral nutrition, n	6	5	11	1	0.47
Parenteral nutrition, n	0	0	0	0	1
ICU survival, n (%)	25 (89)	34 (92)	30 (77)	5 (83)	0.28
28-day survival, n (%)	24 (86)	30 (81)	23 (59)	5 (83)	0.05
90-day survival, n (%)	22 (79)	28 (76)	23 (59)	5 (83)	0.25

*p* < 0.05: $ versus sepsis; € versus septic shock; % versus other shock. BMI: Body Mass Index; SOFA: Sequential Organ Failure Assessment; APACHE II: Acute Physiology and Chronic Health Evaluation; IDDM: insulin-dependent diabetes mellitus; NIDDM: non-insulin-dependent diabetes mellitus; chronic arterial hypertension: blood pressure >140/90 mmHg (ESC); CRF: chronic renal failure—glomerular filtration rate < 60 mL/min/1.73 m^2^ (MDRD).

**Table 3:** Biological data in the different patient groups.

Biological Data	ICU Control Group (n = 28)	Sepsis (n = 37)	Septic Shock (n = 39)	Other Shock (n = 6)	*p* Value
Lactate (mEq/L)	1.2 ( [0.8–1.7]	0.9 [0.7–1.4]	1.6 [1.2–2.4] $	3.1 [1.9–6.6] $	0.001
Glycemia (mg/dL)	142 [125–157]	149 [109–227]	164 [123–183]	181 [140–187]	0.52
Hemoglobin (g/dL)	12.2 [10.0–14.4]	11.2 [10.1–13.7]	10.1 [9.2–11.7] £ $	10.4 [9.6–11.0] £	0.007
White blood cells (10.3/mm^3^)	13.1 [11.4–15.9]	11.4 [7.8–15.2]	17.4 [10.6–24.5]	21.4 [19.9–29.7] £	0.018
Platelets (10.3/mm3)	223 [184–281]	235 [183–311]	230 [142–370]	232 [176–276]	0.94
C-reactive protein (mg/dL)	19 [9–52] €	109 [64–208] % £	222 [118–297]	15 [5–26] €	<0.001
Bilirubin (mg/dL)	0.5 [0.4–0.8]	0.6 [0.4–1.0]	0.7 [0.5–2.1]	0.6 [0.3–1.1]	0.29
BUN (mg/dL)	17.7 [11.7–24.3] €	19.6 [13.5–33.6] €	32.2 [22.9–57.4]	25.7 [15.9–42.9]	<0.001
Creatinin (mg/dL)	0.8 [0.7–0.9] €	0.7 [0.7–1.3] €	1.5 [0.9–2.8]	1.3 [0.8–2.2]	<0.001
Albumin (g/L)	32 [28–35] €	30 [27–33]	27 [22–31]	29 [23–30]	0.037

*p* < 0.05: $ versus sepsis; £ versus ICU control group; € versus septic shock; % versus other shock. Results are presented as median values [ 25–75% percentiles].

**Table 4:** Treatment and hemodynamic variables in the different patient groups.

Treatment and Parameters	ICU Control Group (n = 28)	Sepsis (n = 37)	Septic Shock (n = 39)	Other Shock (n = 6)	*p* Value
Midazolam (mg/h)	0 [0–2]	0 [0–0]	1.5 [0–3.3] $	1 [0–4]	0.036
Morphine (mg/h)	0 [0–2]	0 [0–0]	0 [0–2]	1 [0–2]	0.22
Dobutamine (μg/kg/min)	0 [0–0]	0 [0–0]	0 [0–0]	1 [0–4] $ £ €	<0.001
Norepinephrin (μg/kg/min)	0 [0–0]	0 [0–0]	0.2 [0.07–0.25] $ £	0.14 [0.06–0.2] $ £	<0.001
PaO_2_/FiO_2_	250 [183–299]	175 [135–250]	196 [143–306]	190 [164–279]	0.22
Heart rate (beat/min)	73 [61–92]	101 [79–111] £	97 [85–109] £	100 [75–112]	0.001
Mean arterial pressure (mmHg)	85 [75–93]	82 [75–92]	69 [64–74] $ £	65 [61–71] £ $	<0.001
Temperature (° Celcius)	36.6 [35.7–37.1]	36.8 [36.3–37.1]	36.7 [36.4–37.7]	35.1 [34.5–35.8] € $	0.007

*p* < 0.05: $ versus sepsis; £ versus ICU control group; € versus septic shock; Results are presented as median values (25–75% percentiles).

**Table 5:** Comparisons of the area under the curve (AUC) values for several metabolic products in the four patient groups.

	ICU Control Group (n = 26)	Sepsis (n = 31)	Septic Shock (n = 37)	Other Shock (n = 5)	*p* Value
**Phenylalanine**	0.00803 [0.0068–0.00967] £ *	0.0103 [0.00822–0.0143]	0.0113 [0.00916–0.0142]	0.986 [0.00744–0.0124]	0.004
**Lactate**	1.357 [1.062–1.821]	1.2 [0.869–1.605) £	1.616 [1.218–2.08]	2.12 5 [1.181–2.987]	0.017
**Carnitine**	0.06 [0.0453–0.0916] £	0.0634 [0.0353–0.0785] £	0.1 [0.0655–0.164]	0.0958 [0.0929–0.145]	0.016
**Urea**	0.0228 [0–0.0442] £	0.025 [0–0.0414] £	0.0752 [0.0289–0.156]	0.0647 [0.0153–0.124]	<0.001
**Myo-inositol**	0.0085 [0–0.0153] £	0.00838 [0–0.0222] £	0.0216 [0.00864–0.0421]	0.0148 [0.00819–0.0292]	0.001
**Creatinine**	0.0314 [0.0282–0.0424] ** £	0.0313 [0.0257–0.0485] £	0.0708 [0.041–0.105]	0.0783 [0.0563–0.123]	0.002
**Creatinine (singulet)**	0.0475 [0.0475–0.0554] £	0.0463 [0.0399–0.0615] ** £	0.0863 [0.0583–0.149]	0.101 [0.0752–0.16]	<0.001
**Creatine**	0.0189 [0.0113–0.0259] £ *	0.0382 [0.0169–0.0731]	0.0645 [0.0287–0.136]	0.0245 [0.0128–0.0527]	<0.001
**Creatine**	0.0183 [0.0121–0.0219] £	0.029 [0.0161–0.0622] £	0.0458 [0.0319–0.103]	0.0145 [0.012–0.0415] £	<0.001
**Citrate**	0.0314 [0.0234–0.0355] *	0.0227 [0.018–0.0297] **	0.0277 [0.0201–0.0343]	0.037 [0.0319–0.0503]	0.004
**Pyruvate**	0.0377 [0.0282–0.0533]	0.0334 [0.0246–0.0487] £	0.0509 [0.0395–0.0671]	0.047 [0.0296–0.102]	0.015
**2-hydroxyisovalerate**	0.00529 [0−0.0851] **	0.00862 [0−0.0147]	0.00841 [0.00474–0.0185]	0.0714 [0.0446–0.0854] * £	0.002
**Mannitol**	0.0213 [0.00938–0.122] £	0.00882 [0.00513–0.017] £	0.0958 [0.024–0.313]	0.00918 [0.00663–0.0446] £	<0.001
Glycerol	0.177 [0.135–0.229]	0.169 [0.151–0.205]	0.169 [0.137–0.212]	0.2 [0.153–0.211]	0.984
TMAO	0.0554 [0.00988–0.117]	0.0518 [0.0372–0.0851]	0.0847 [0.051–0.124]	0.121 [0.0733–0.22]	0.205
Ethanol	0.00498 [0–0.0111]	0.00758 [0.00578–0.0184]	0.00766 [0.00213–0.0124]	0.0134 [0.00172–0.0232]	0.173
3-hydroxyxisobutyrate	0.0105 [0.00732–0.014]	0.0125 [0.0093–0.0175]	0.0161 [0.0117–0.0212]	0.0135 [0.0097–0.0196]	0.166
Isoleucine	0.0242 [0.0178–0.0312]	0.0301 [0.0212–0.0396]	0.0257 [0.0185–0.0325]	0.0211 [0.0202–0.0307]	0.33
Tyrosine	0.0196 [0.0151–0.0226]	0.0198 [0.0159–0.027]	0.0187 [0.0147–0.0278]	0.0209 [0.0132–0.0271]	0.96
Glucose	0.583 [0.492–0.657]	0.664 [0.559–0.969]	0.699 [0.486–0.791]	0.833 [0.613–0.914]	0.078
Choline	0.0177 [0.0126–0.0232]	0.0229 [0.0146–0.0276]	0.0191 [0.0114–0.0279]	0.0248 [0.0226–0.0306]	0.242
Acetylcarnitine	0.00873 [0.00764–0.0173]	0.0136 [0.00405–0.0192]	0.0192 [0.00708–0.0327)	0.015 [0.013–0.0293]	0.115
Malonate	0 [0–0.00426]	0.00313 [0–0.0112]	0 [0–0.0087]	0 [0–0.00497]	0.465
Glutamine	0.183 [0.134–0.222]	0.262 [0.184–0.28]	0.18 [0.119–0.206]	0.181 [0.132–0.206]	0.337
3-hydroxybutyrate	0.0181 [0–0.0374]	0.018 [0–0.0545]	0.0183 [0–0.0545]	0.0145 [0–0.0508]	0.993
Acetoacetate	0.0136 [0.0103–0.0295]	0.0199 [0.00971–0.0385]	0.0163 [0.00923–0.0256]	0.0195 [0.00916–0.0306]	0.864
Acetone	0 [0−0.003]	0.00201 [0–0.046]	0 [0−0.00333]	0 [0−0.00136]	0.338
Acetate	0.0209 [0.172–0.0283]	0.0189 [0.014–0.0241)	0.0188 [0.0165–0.0252]	0.0244 [0.0171–0.0507]	0.327
Alanine	0.17 [0.143–0.259]	0.158 [0.123–0.199)	0.173 [0.126–0.208)	0.22 [0.196–0.342]	0.163
Peak 1.44	0 [0–0] £	0 [0–0]	0 [0–0.0154]	0 [0–0.0331]	0.03
Peak 1.41	0 [0–0] £	0 [0–0.0176]	0.0097 [0.00307–0.0638]	0 [0–0] £	<0.001
3-hydroxybutyrate	0.0635 [0.0354–0.147]	0.0536 [0.0357–0.195]	0.0722 [0.0282–0.182]	0.0461 [0.0318–0.011]	0.961
Propylene glycol	0 [0−0.142]	0.0068 [0–0.059]	0.0272 [0−0.201)	0 [0−0.332]	0.258
Valine	0.101 [0.0844–0.116]	0.114 [0.0931–0.146]	0.0877 [0.0733–0.163]	0.102 [0.0977–0.124]	0.343
Betaine	0.0201 [0.0132–0.0283]	0.0248 [0.0178–0.0396]	0.023 [0.0127–0.0301]	0.0231 [0.0204–0.0362]	0.149
Leucine	0.0826 [0.0675–0.103]	0.116 [0.0874–0.134]	0.0883 [0.0597–0.141]	0.108 [0.0858–0.129]	0.06
Hydroxybutyrate	0.0467 [0.0317–0.0629]	0.0645 [0.0481–0.0919]	0.0571 [0.0333–0.0985]	0.0714 [0.0446–0.0854]	0.186

*p* < 0.05: * versus sepsis; versus ICU control group; £ versus septic shock; ** versus other shock; Bold texts indicate metabolites with significant difference from the other metabolites.

**Table 6:** Correlations between metabolites, length of ICU stay and mortality.

Metabolites	Length of ICU Stay	ICU Mortality	28-Day Mortality	90-Day Mortality
Phenylalanine	r_s_ = −0.18	r_s_ = 0.06	r_s_ = −0.08	r_s_ = −0.05
	*p* = 0.07	*p* = 0.55	*p* = 0.44	*p* = 0.61
Mannitol	r_s_ = −0.07	r_s_ = −0.1	r_s_ = −0.13	r_s_ = −0.15
	*p* = 0.53	*p* = 0.35	*p* = 0.22	*p* = 0.14
Carnitine	r_s_ = 0.03	r_s_ = −0.1	r_s_ = −0.02	r_s_ = 0.05
	*p* = 0.79	*p* = 0.41	*p* = 0.87	*p* = 0.65
Lactate	r_s_ = −0.13	r_s_ = −0.13	r_s_ = −0.2	r_s_ = −0.17
	*p* = 0.20	*p* = 0.2	*p* = 0.04	*p* = 0.1
Urea	r_s_ = −0.13	r_s_ = −0.10	r_s_ = −0.08	r_s_ = −0.02
	*p* = 0.19	*p* = 0.31	*p* = 0.42	*p* = 0.88
Myo-inositol	r_s_ = −0.12	r_s_ = −0.03	r_s_ = −0.06	r_s_ = 0.06
	*p* = 0.25	*p* = 0.76	*p* = 0.55	*p* = 0.54
Creatinine	r_s_ = −0.15	r_s_ = −0.08	r_s_ = −0.1	r_s_ = −0.02
	*p* = 0.14	*p* = 0.42	*p* = 0.34	*p* = 0.85
Creatine	r_s_ = −0.17	r_s_ = 0.07	r_s_ = 0.04	r_s_ = 0.007
	*p* = 0.1	*p* = 0.52	*p* = 0.73	*p* = 0.94
Pyruvate	r_s_ = −0.04	r_s_ = −007	r_s_ = −0.07	r_s_ = −0.03
	*p* = 0.7	*p* = 0.95	*p* = 0.49	*p* = 0.76

r_s_: Spearman coefficient; *p*: *p*-value.

## Data Availability

The datasets analyzed during the current study are available from the corresponding author upon reasonable request. The data are not publicly available due to privacy or ethical restrictions.

## Supplementary Materials

The following supporting information can be downloaded at: https://www.mdpi.com/article/10.3390/metabo13040523/s1, Figure S1: Spectra of metabolites between ICU groups of patients, Figure S2: Heatmaps of metabolites.
