# Supplementary material for: Serum Metabolomic Profiles in Critically Ill Patients with Shock on Admission to the Intensive Care Unit
Source: Metabolites. 2023 Apr 5;13(4):523. doi: 10.3390/metabo13040523 (PMC10144913; doi:10.3390/metabo13040523)
Supplement: Supplementary file 1 [file metabolites-13-00523-s001.zip › Fig S1 Heatmap_usi .pdf]

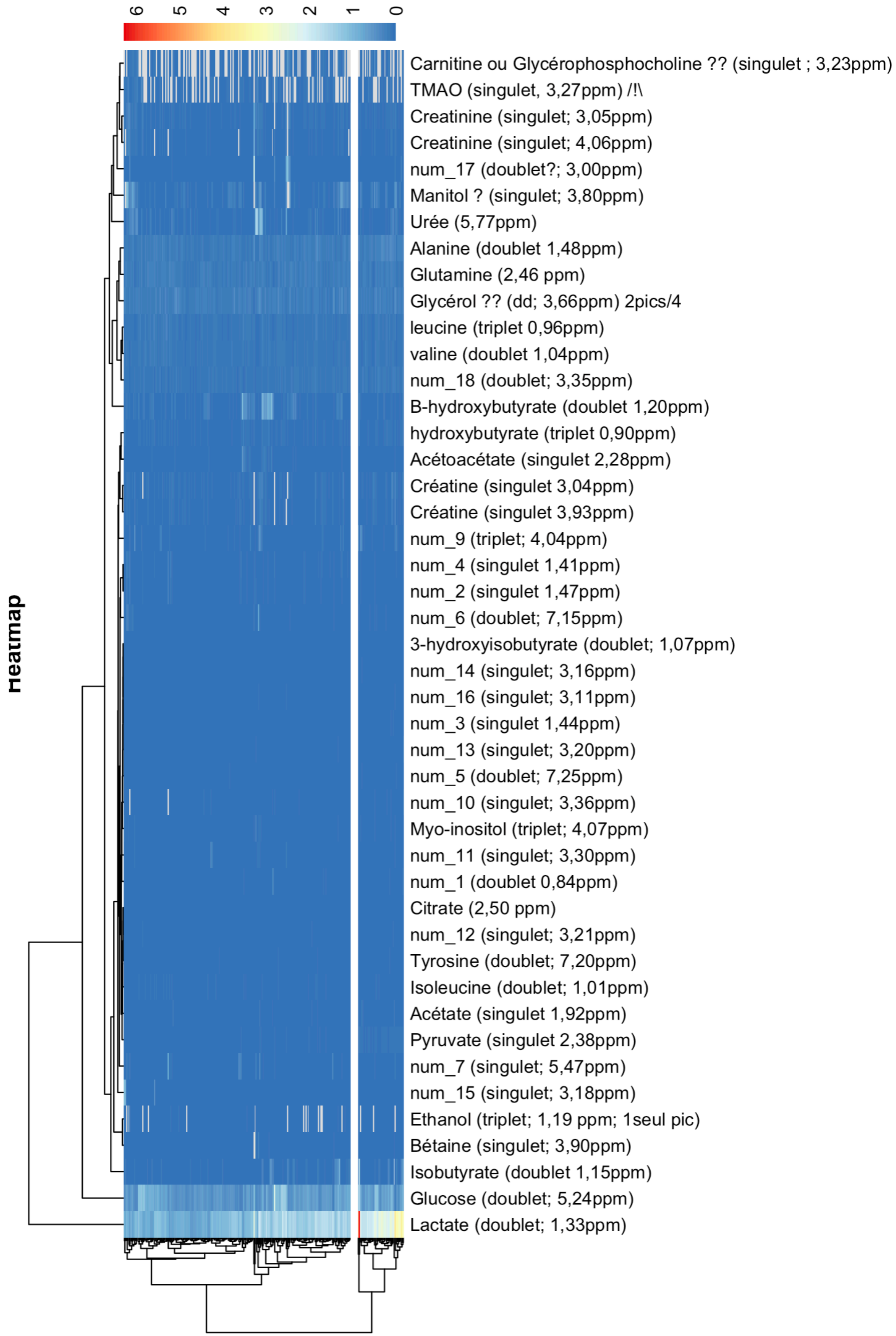

Heatmap
6
5
4
3
2
1
0
Carnitine ou Glycérophosphocholine ?? (singulet ; 3,23ppm)
TMAO (singulet, 3,27ppm) /!\
Creatinine (singulet; 3,05ppm)
Creatinine (singulet; 4,06ppm)
num_17 (doublet?; 3,00ppm)
Manitol ? (singulet; 3,80ppm)
Urée (5,77ppm)
Alanine (doublet 1,48ppm)
Glutamine (2,46 ppm)
Glycérol ?? (dd; 3,66ppm) 2pics/4
leucine (triplet 0,96ppm)
valine (doublet 1,04ppm)
num_18 (doublet; 3,35ppm)
B-hydroxybutyrate (doublet 1,20ppm)
hydroxybutyrate (triplet 0,90ppm)
Acétoacétate (singulet 2,28ppm)
Créatine (singulet 3,04ppm)
Créatine (singulet 3,93ppm)
num_9 (triplet; 4,04ppm)
num_4 (singulet 1,41ppm)
num_2 (singulet 1,47ppm)
num_6 (doublet; 7,15ppm)
3-hydroxyisobutyrate (doublet; 1,07ppm)
num_14 (singulet; 3,16ppm)
num_16 (singulet; 3,11ppm)
num_3 (singulet 1,44ppm)
num_13 (singulet; 3,20ppm)
num_5 (doublet; 7,25ppm)
num_10 (singulet; 3,36ppm)
Myo-inositol (triplet; 4,07ppm)
num_11 (singulet; 3,30ppm)
num_1 (doublet 0,84ppm)
Citrate (2,50 ppm)
num_12 (singulet; 3,21ppm)
Tyrosine (doublet; 7,20ppm)
Isoleucine (doublet; 1,01ppm)
Acétate (singulet 1,92ppm)
Pyruvate (singulet 2,38ppm)
num_7 (singulet; 5,47ppm)
num_15 (singulet; 3,18ppm)
Ethanol (triplet; 1,19 ppm; 1seul pic)
Bétaine (singulet; 3,90ppm)
Isobutyrate (doublet 1,15ppm)
Glucose (doublet; 5,24ppm)
Lactate (doublet; 1,33ppm)
