# Supplementary figures and images for: Serum Metabolomic Profiles in Critically Ill Patients with Shock on Admission to the Intensive Care Unit
Source: Metabolites. 2023 Apr 5;13(4):523. doi: 10.3390/metabo13040523 (PMC10144913; doi:10.3390/metabo13040523)

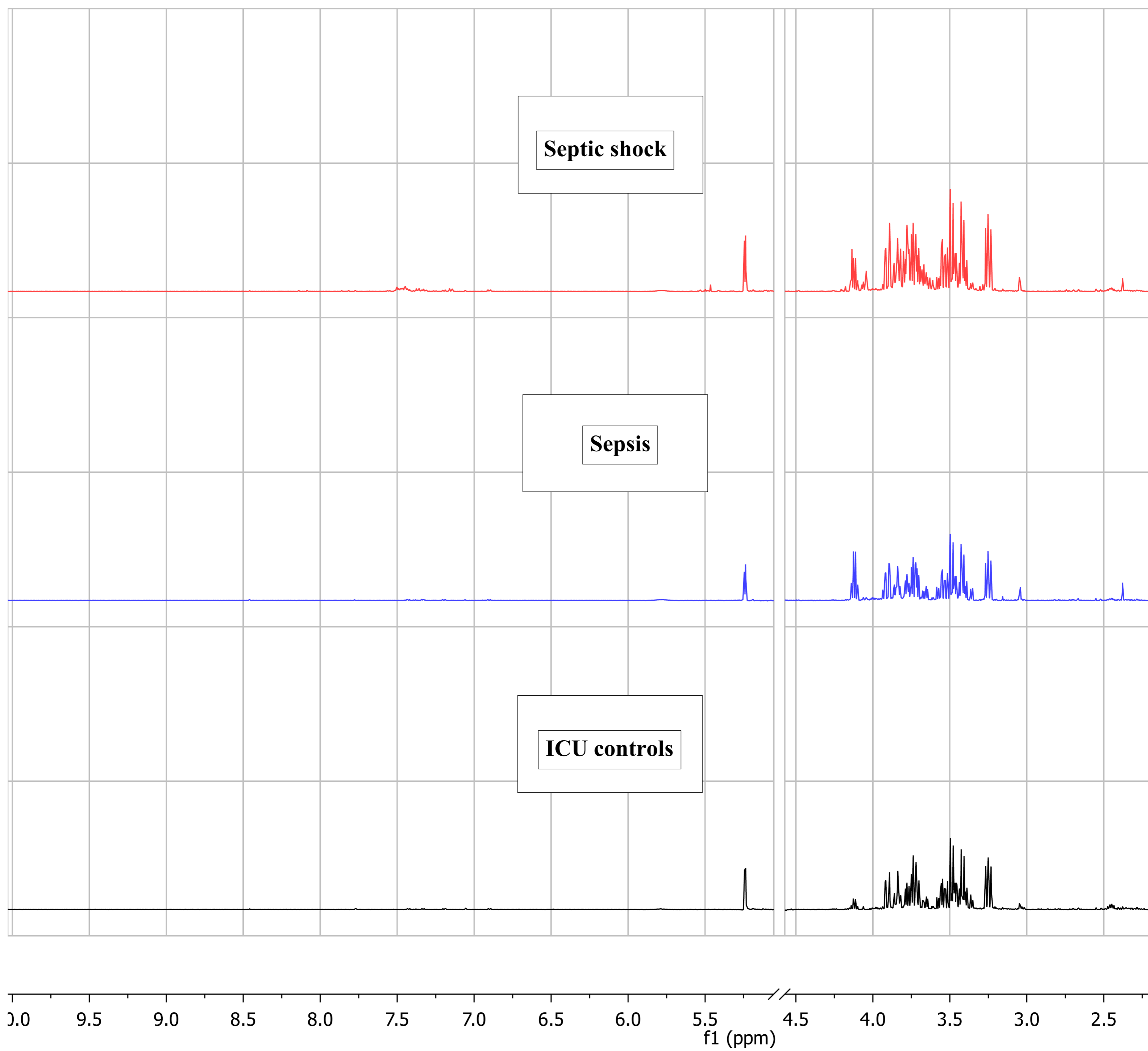

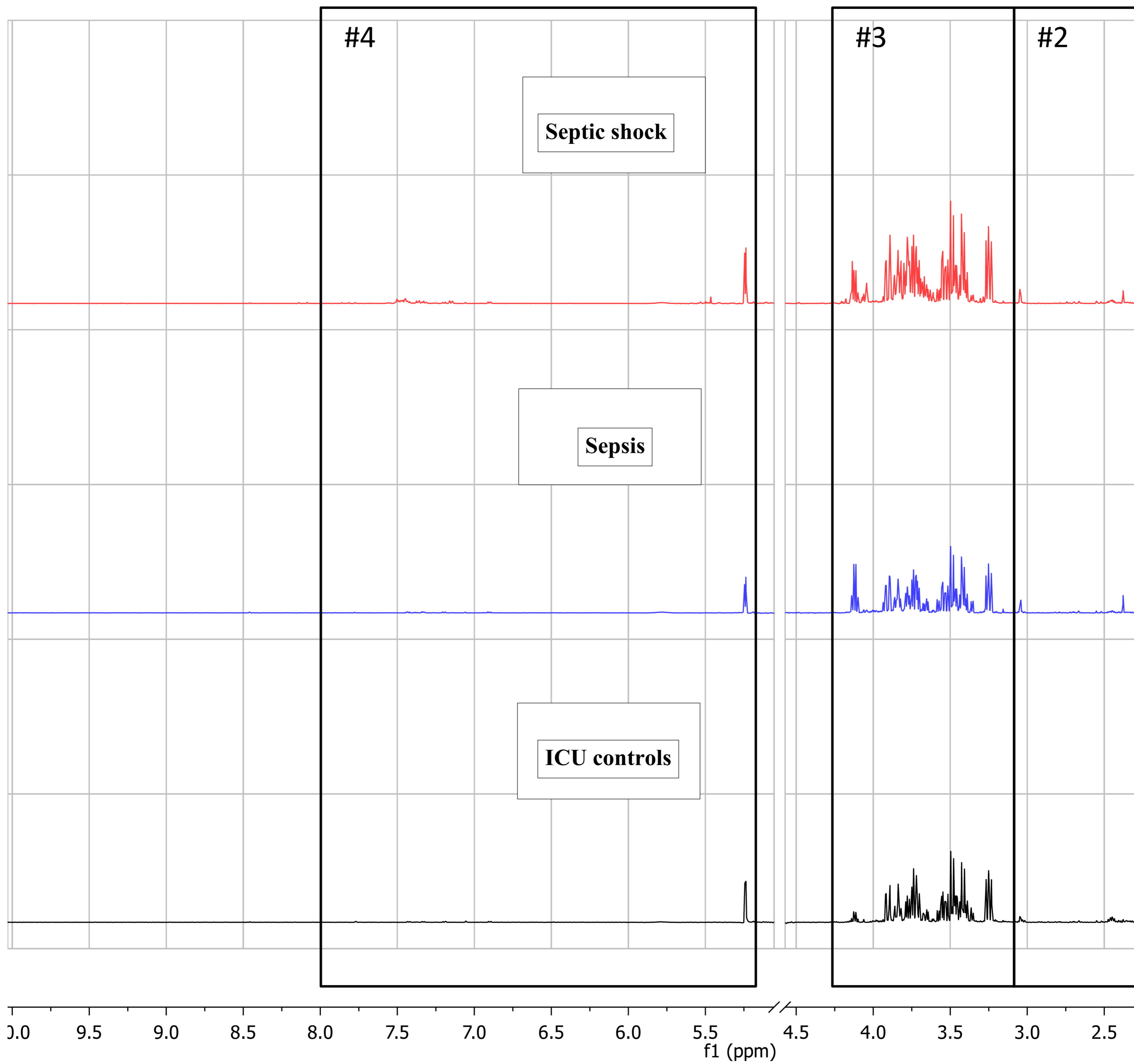

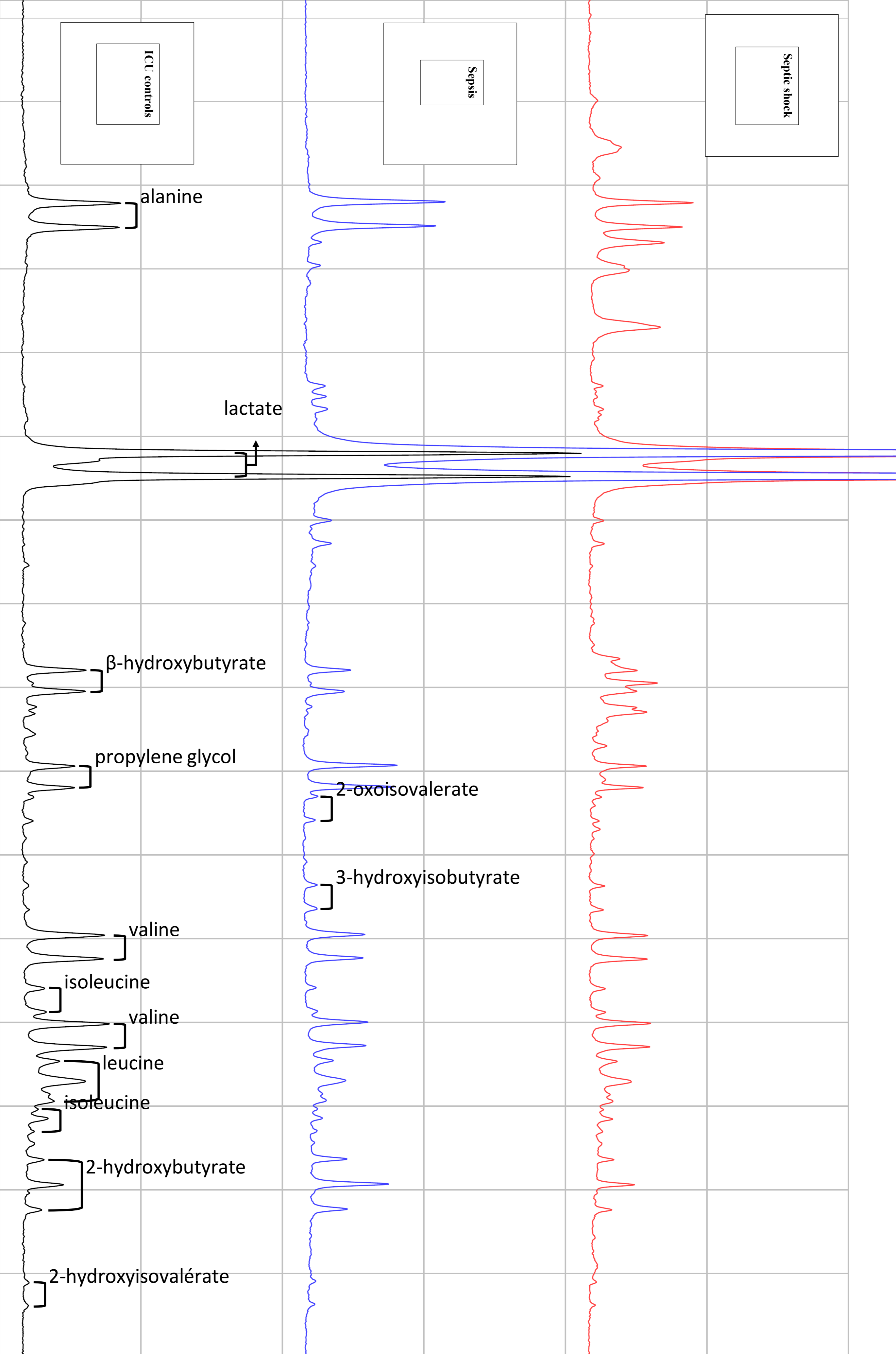

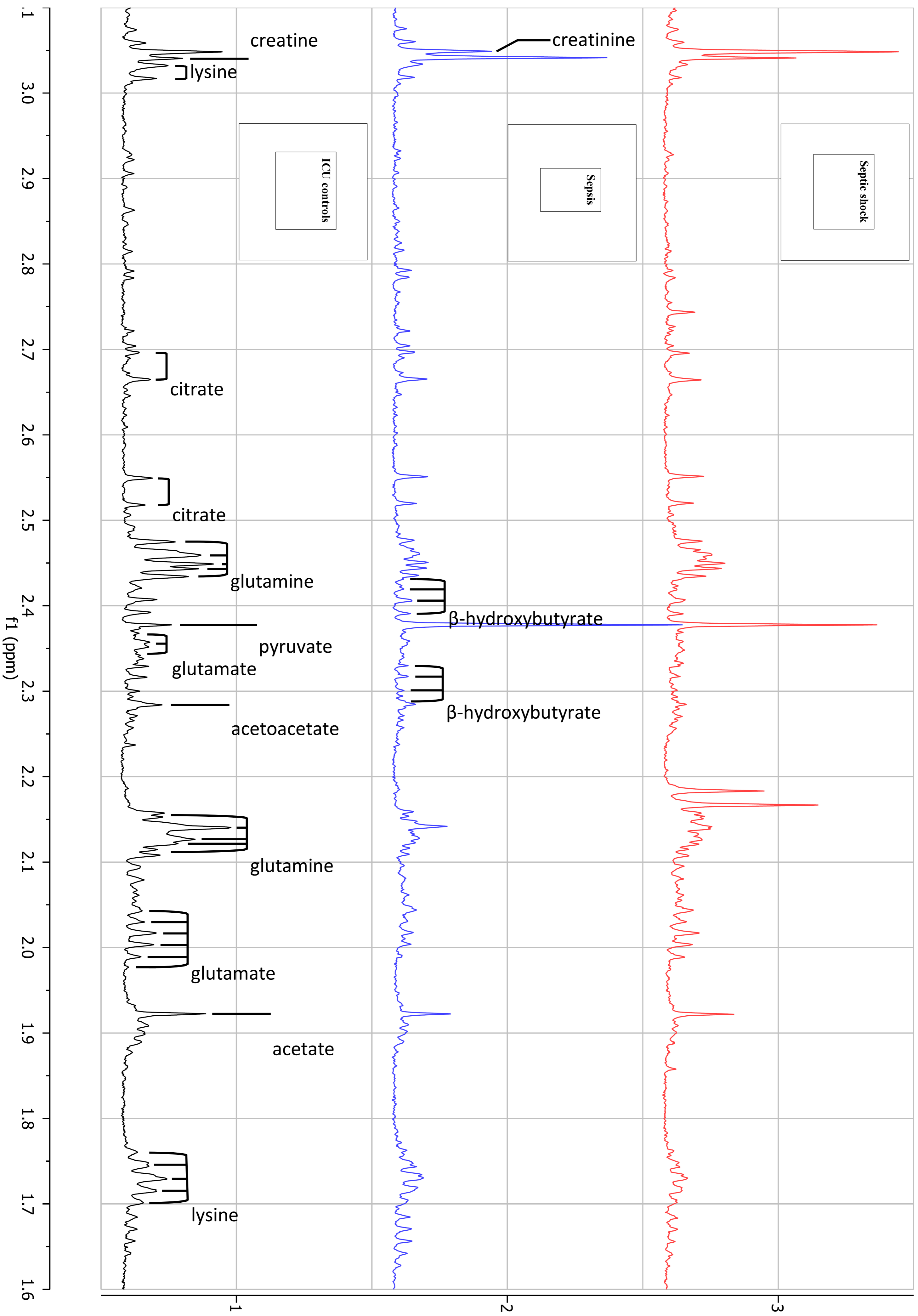

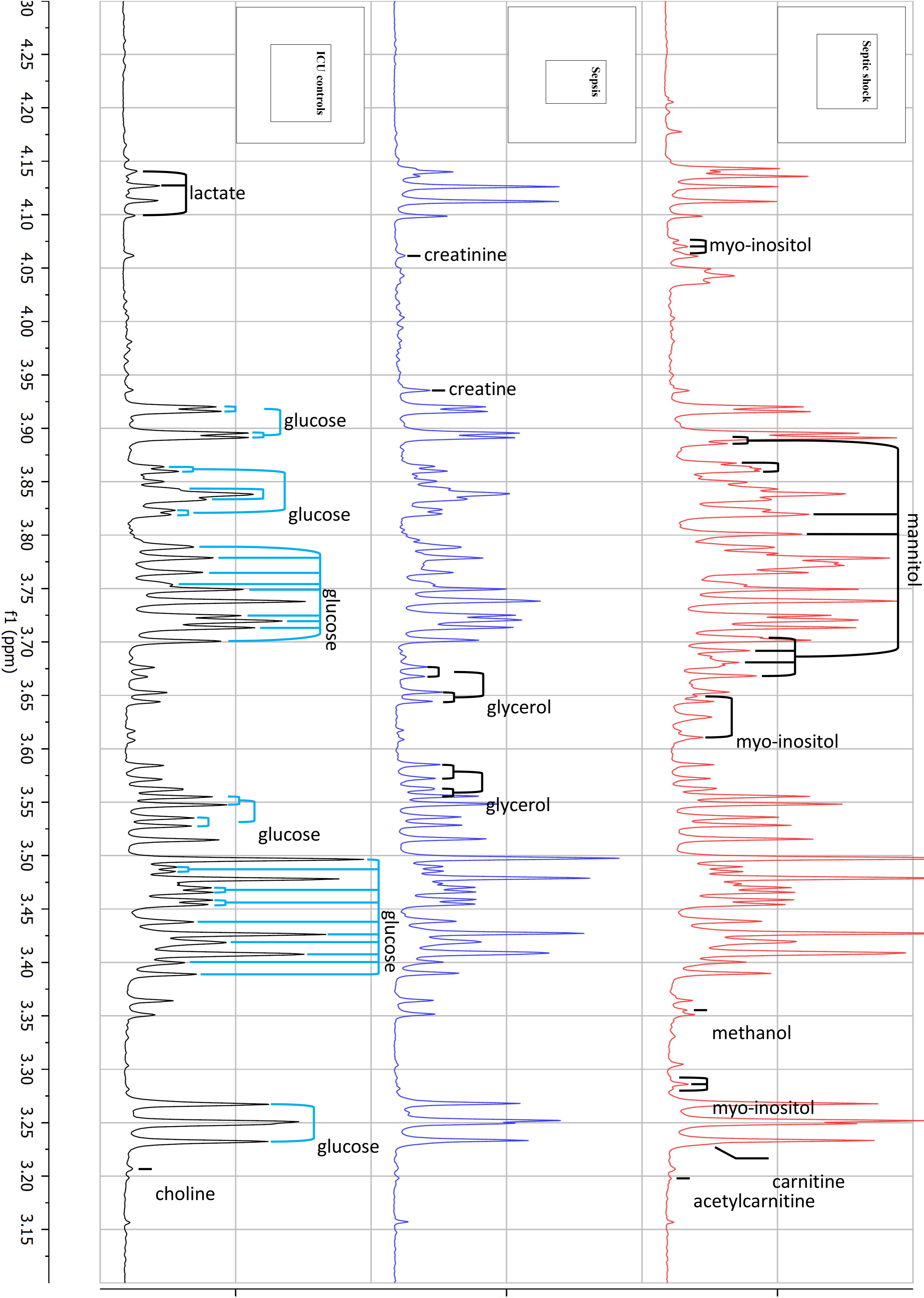

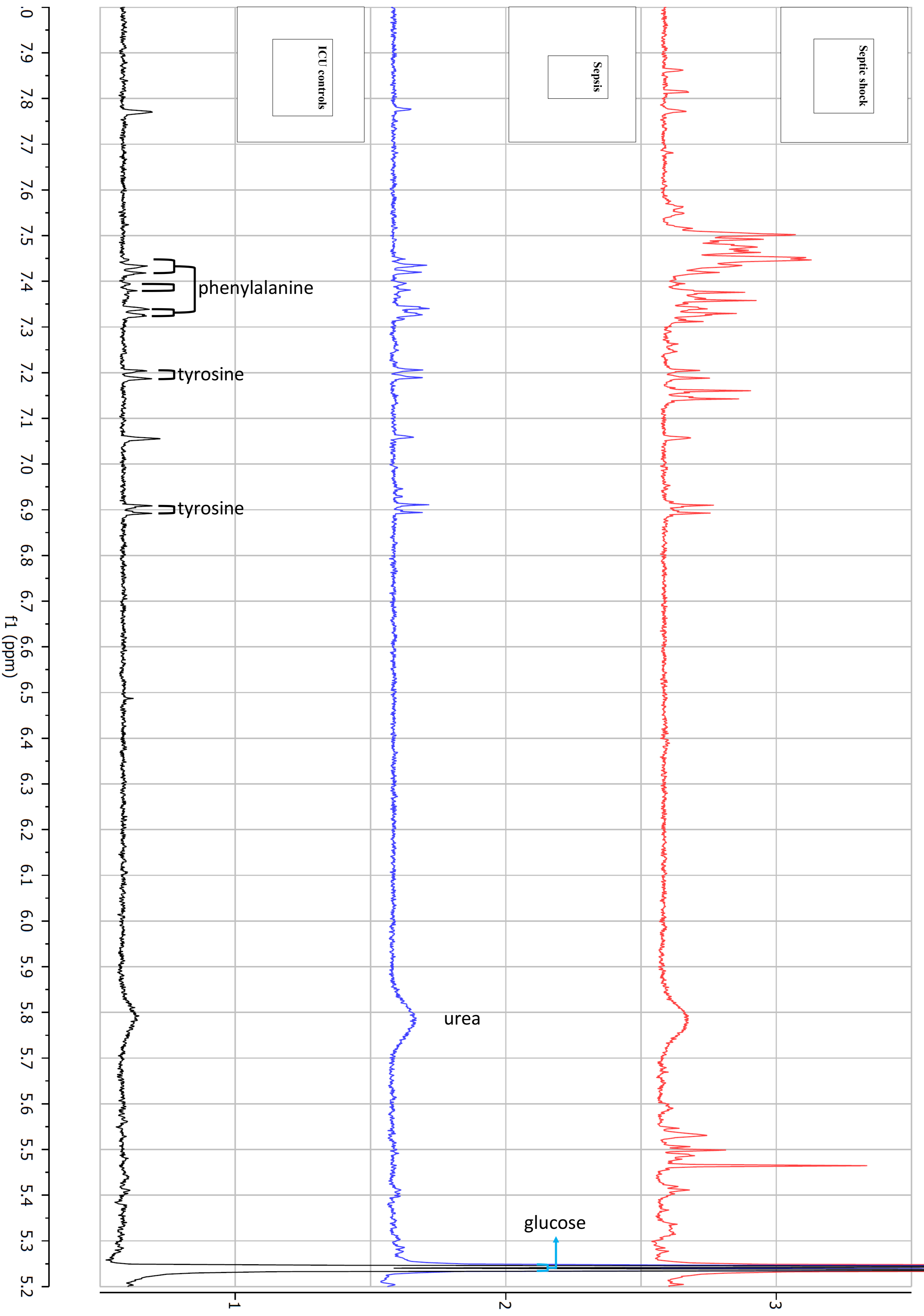

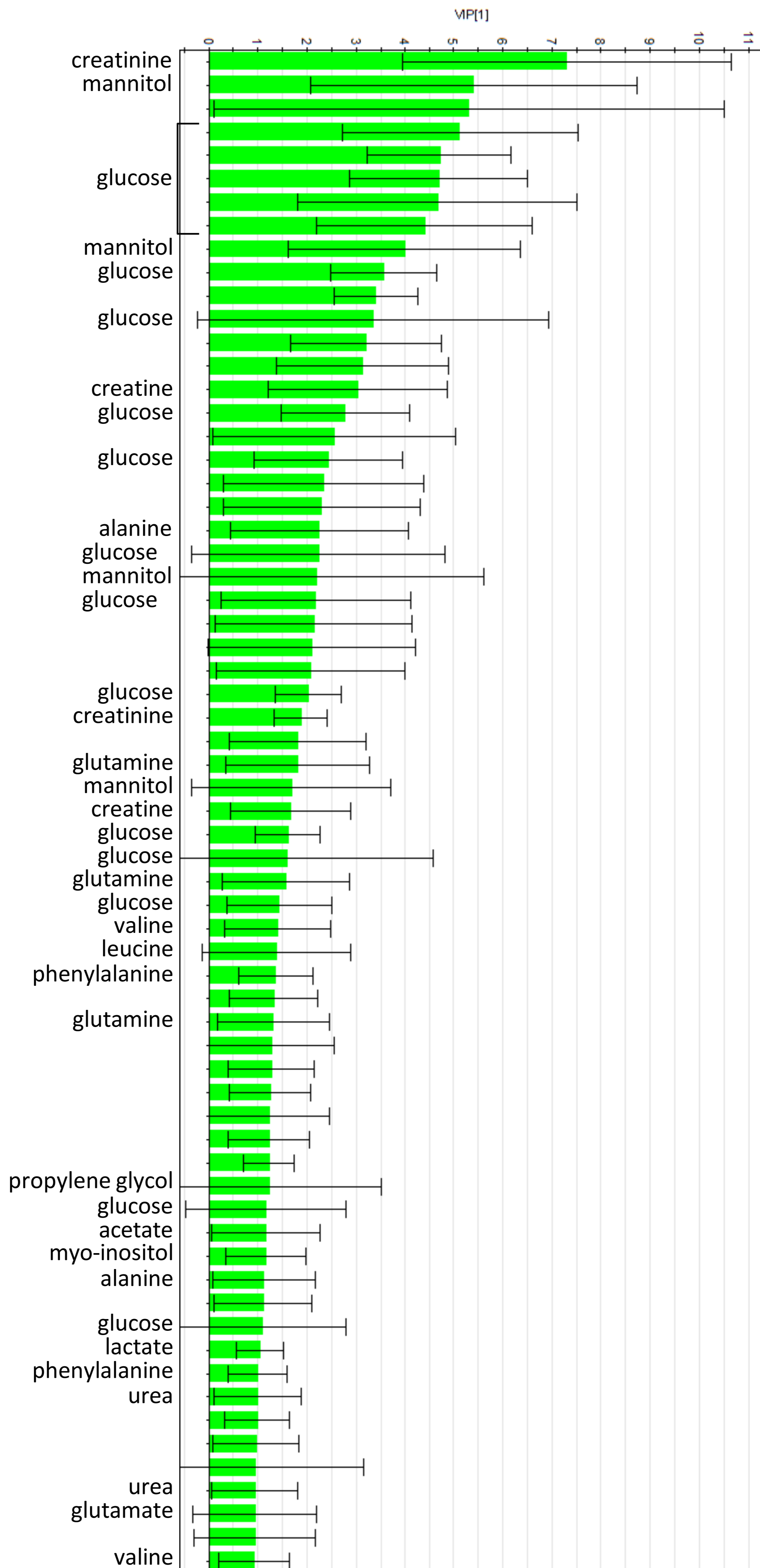

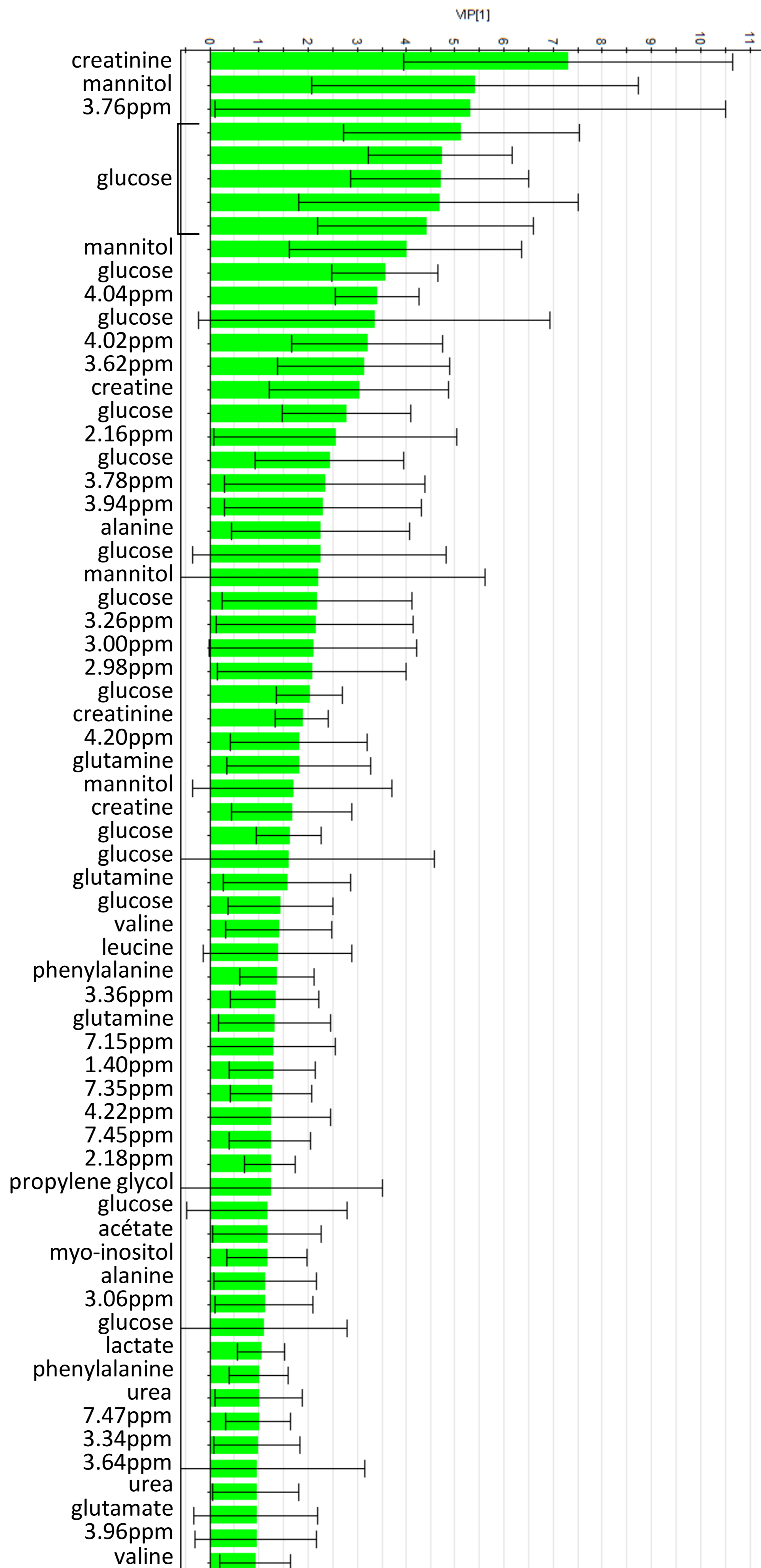

Supplement: Supplementary file 1 [file metabolites-13-00523-s001.zip › Fig S2 Spectra .pdf]
